# Coagulation Disorders and Thrombosis in COVID-19 Patients and a Possible Mechanism Involving Endothelial Cells: A Review

**DOI:** 10.14336/AD.2021.0704

**Published:** 2022-02-01

**Authors:** An-tian Chen, Chen-yu Wang, Wen-ling Zhu, Wei Chen

**Affiliations:** ^1^Department of Cardiology, Peking Union Medical College Hospital, Chinese Academy of Medical Sciences and Peking Union Medical College, Beijing 100730, China; ^2^Department of Computer Science, University of Texas at Austin, Austin, TX, USA

**Keywords:** COVID-19, SARS-CoV-2, coagulation, thrombosis, endothelial cells

## Abstract

Coronavirus disease 2019 (COVID-19) is still an ongoing pandemic worldwide. COVID-19 is an age-related disease with a higher risk of organ dysfunction and mortality in older adults. Coagulation disorders and thrombosis are important pathophysiological changes in COVID-19 infection. Up to 95% of COVID-19 patients have coagulation disorders characterized by an elevated D-dimer, a prolonged prothrombin time, a low platelet count and other laboratory abnormalities. Thrombosis is found in critical cases with an increased risk of death. Endothelial cells are prone to be affected by the novel SARS-CoV-2 and express angiotensin-converting enzyme 2. The evidence, such as the presence of the virus, has been identified, leading to the inflammation and dysfunction. Endothelial cell activation and dysfunction play a pivotal role in the hypercoagulation status in COVID-19 patients. In addition to the direct exposure of subendothelial tissue to blood, Weibel-Palade bodies within the endothelium containing coagulants can be released into the circulation. Endothelial nitric oxide synthase may be impaired, thus facilitating platelet adhesion. Moreover, anti-β2-glycoprotein I antibodies may also contribute to the coagulopathy in COVID-19 by inducing the upregulation of proinflammatory mediators and adhesion molecules. To conclude, coagulation disorders and thrombosis are vital and predict a poor outcome in COVID-19 patients, especially in severe cases. Endothelial cell activation and dysfunction may play an important role in causing clot formation. More basic and clinical research is warranted to further our understanding of the role of coagulopathy and their possible mechanism in COVID-19 patients.

Coronavirus disease 2019 (COVID-19) was first reported on December 31, 2019, in Wuhan city, China [1]. On March 11, it was announced to be a pandemic of international concern by the World Health Organization. The pathogen was first called 2019 novel coronavirus (2019-nCoV) [2], but then it was officially named severe acute respiratory syndrome coronavirus (SARS-CoV-2) by the Coronaviridae Study Group (CSG) of the International Committee on the Taxonomy of Viruses [3]. COVID-19 patients are often observed to have coagulation disorders, and the coagulation disorders have become a remarkable characteristic in need of special attention. Here, we summarize the knowledge regarding coagulation disorders in COVID-19 patients and the possible mechanism of thrombosis focusing on endothelial cells, and we provide a brief overview of anticoagulation treatment strategies.

## 1. Coagulation disorders

Clinically, COVID-19 patients are mostly characterized by respiratory symptoms and disorders. However, coagulation disorders are quite common in COVID-19 patients (Table 1) and sometimes result in thromboses in respiratory, cardiovascular, and venous systems. Coagulopathy not only causes vessel occlusion but also predicts a poor outcome in COVID-19 patients. Additionally, COVID-19 is clearly an age-related disease, and older people are at a higher risk [4]. The mortality rate of elderly patients is 15% higher than that of young patients [5].

**Table 1:** Clinical Findings of Coagulation Disorders in COVID-19 Patients.

Source	Country	Sample Size	Study Design/type	Coagulation Tests	Results
Guan et al [7]	China	1099	Multicenter retrospective study	PLT and D-Dimer	Lower PLT and elevated D-dimer (≥0.5 mg/L) levels were more profound and frequent in severe patients (137,500/mm^3^ vs 172,000/mm^3^, 59.6% vs 43.2%) and in the presence of a composite primary endpoint (156,500/mm^3^ vs 169,000/mm^3^, 69.4% vs 44.2%).
Helms et al [6]	France	150	Multicenter prospective cohort study	PLT, APTT, PT, INR, D-dimer, fibrinogen, AT, factor V, factor VIII, vWF and LAC	More than 95% of patients had elevated D-dimer and fibrinogen levels; PLT, PT, APTT and antithrombin were within normal range mostly; vWF activity, vWF antigen and factor VIII were elevated; and LAC were 87.7% (50/57) positive in ICU patients.
Huang et al [8]	China	41	Single-center, prospective study	PLT, PT, APTT and D-dimer	ICU patients had higher PT (12.2 s) and D-dimer levels (2.4 mg/L) on admission than non-ICU patients (10.7 s, 0.5 mg/L, respectively).
Shi et al [11]	China	416	Single-center, retrospective cohort study	PLT	Patients with cardiac injury had lower platelet counts (172 cells?×?10^3^/μL vs 216 cells?×?10^3^/μL).
Tang et al [9]	China	183	Single-center, retrospective study	PT, APTT, AT, fibrinogen, FDP, and D-dimer	Remarkably elevated D-dimer and FDP levels were common in patients who died; a prolonged PT, lower fibrinogen, and AT levels were also found in nonsurvivors.
Xiong et al [24]	China	1105	Meta-analysis of 9 studies	PLT (5 studies), APTT (6), PT (6) and D-dimer (8)	PT and D-dimer levels were remarkably higher in severe patients, while no significant difference was found between severe and mild patients regarding PLT and APTT.
Beyrouti et al [19]	UK	6	Case series	PLT, PT, INR, APTT, fibrinogen, D-dimer, and aPLs	D-dimer levels were all higher than 1000 µg/L; IgG and IgM aCL and aβ2GP1 were negative, while 5/6 patients were positive with LAC.
Zhang et al [16]	China	3	Case series	PLT, PT, APTT, fibrinogen, FDP, D-dimer, and aPLs	3 patients were positive for aCL IgA as well as aβ2GPI IgA and IgG.
Harzallah et al [20]	France	56	Case series	aCL, aβ2GPI and LAC	LAC was positive in 25 cases (45%), while aCL or aβ2GPI were positive only in 5 cases (10%, 3 associated with LAC).
Escher et al [22]	Switzerland	1	Case report	D-dimer, aPLs, vWF and factor VIII	D-dimer, IgM aCL, IgM aβ2GPI, vWF activity, vWF antigen and factor VIII were elevated.

Abbreviations: PLT, platelet count; PT, prothrombin time; APTT, activated partial thromboplastin time; aPLs, antiphospholipid antibodies; aCL, anticardiolipin antibodies; aβ2GPI: anti-β2-glycoprotein-1 antibodies; AT, antithrombin activity; LAC, lupus anticoagulant; FDP, fibrin degradation product. Note: Data listed in the table are median levels.

### 1.1 Elevated D-dimer, prolonged prothrombin time and decreased platelet count

Coagulation disorders are common and have been noted in several studies, and an elevated D-dimer has been a significant finding in COVID-19 patients. In a multicenter prospective cohort study, more than 95% of patients had elevated D-dimer and fibrinogen levels [6]. Specifically, an elevated D-dimer (≥0.5 mg/L) was more frequent in severe patients in a study that included 1099 patients [7]. A group of 41 COVID-19 patients including 13 patients in an intensive care unit (ICU) and 28 non-ICU patients were reported to have leukopenia and lymphopenia on admission and higher D-dimer levels (2.4 mg/L, IQR 0.6-14.4 mg/L) in ICU patients compared to 0.5 mg/L, and IQR 0.3-0.8 mg/L in non-ICU patients [8]. COVID-19 patients who suffered from stroke in the UK were all found to have elevated D-dimer levels (≥1000 μg/L). Although an elevation in D-dimer levels is common in COVID-19, this elevation can also be observed in infectious diseases caused by other pathogens, especially in ICU patients. Whether the elevation is due to a specific mechanism of COVID-19 or is due to the degree of severity of the disease in these patients is still unknown and remains an important question to be answered.

Prothrombin time (PT) is elevated in COVID-19 patients also but not as significantly elevated as D-dimer. PT may predict a poor outcome because patients who are in the ICU (12.2 s, IQR 11.2-13.4) or who have died of COVID-19 (15.6 s, 14.4-16.3) had a longer PT than non-ICU patients (10.7 s, IQR 9.8-12.1) and surviving patients (13.6 s, 13.0-14.3)[8, 9]. However, as commented by Levi and his colleagues, small changes in PT may be missed when the international normalized ratio (INR) is used instead [10].

Thrombocytopenia is another feature of coagulation disorders. Approximately 5% of infected patients had platelet counts lower than 100×10^9^/L, and a lower platelet count was more frequent and notable in ICU patients with platelet counts and proportions of 137.5×10^9^/L (IQR 99-179.5×10^9^/L) and 8%, respectively, in contrast with that in non-ICU patients (172×10^9^/L, IQR 139-212×10^9^/L and 4%, respectively) [7, 10]. For patients with cardiac injuries, the platelet count was 172 cells?×?10^3^/μL (median) compared to 216 cells?×10^3^/μL (median) in patients without cardiac injuries [11]. Coagulation disorders in COVID-19 patients resemble the traits of disseminated intravascular coagulation (DIC), but COVID-19 is unique because patients have a much higher D-dimer level and less severe thrombocytopenia compared to patients with DIC [10]. The relatively mild decrease in the platelet count may be explained by inflammatory cytokines such as IL-1β and IL-6, which induce increased levels of fibrinogen, and both factors can be generated under inflammatory conditions [12]. Additionally, the mismatched elevated D-dimer levels make sense because of both the rise of fibrinolysis induced by urokinase-type plasminogen activator (u-PA) and the release of plasminogen activators [13]. DIC occurs at a high frequency in severe COVID-19 cases with respiratory failure[9], which increases the risk of multiple-organ failure and even death [14].

However, some studies did not find a significant difference in platelet counts between COVID-19 patients and others, as shown in Table 1. This discrepancy may be caused by the complex bidirectional interactions between platelets and pathogens [15]. Surprisingly, platelets have both direct and indirect effects on pathogens. Platelets directly contribute to pathogen encapsulation and elimination, and can indirectly deal with pathogens by recruiting leukocytes, upregulating pathogen killing by macrophages, enhancing NET (neutrophil extracellular trap) formation, and promoting adaptive immunity. On the other hand, pathogens could alter platelet function by changing the activation process and inducing platelet apoptosis. The platelet count is also altered because of the enhancement of platelet clearance and a decrease in platelet production. Moreover, pathogens impact the host autoimmune and alloimmune response to platelet antigens. This may be because host platelets react more positively and surpass the effect that pathogens have in reducing platelet counts, which results in an insignificant difference in platelet count between COVID-19 patients and others.

### 1.2 Detection of antiphospholipid antibodies

Special attention has also been given to antiphospholipid antibodies (aPLs) after they were reported in severe COVID-19 patients, who had findings of the presence of anticardiolipin IgA antibodies, anti-β2-glycoprotein I IgA antibodies and anti-β2-glycoprotein IgG antibodies [16]. These antibodies are usually utilized to diagnose antiphospholipid syndrome and exist in severe illnesses as well as in certain infections [17]. The presence of aPLs is a risk factor for thrombotic diseases, including stroke, myocardial infarction, pulmonary embolism (PE) and deep vein thrombosis (DVT) [18]. Lupus anticoagulant (LAC), which is another risk factor for thromboembolism, was found in stroke patients with COVID-19 in the UK [19]. Harzallah, Bowles and Helms reported a high presence of LAC in 45% to 91% of patients who were severely ill, were in the ICU or had a high activated partial thromboplastin time (aPTT) [20]. Interestingly, all 8 cases that were positive for either aPLs or LAC from the first two case series were stroke patients [16, 19], while no details about stroke were provided in the third study. Because of their thrombosis-inducing effects, the identification of aPLs and LAC may partly explain the hypercoagulation status and clot formation in some COVID-19 cases. aPLs/LAC are more likely to be positive in stroke patients (8/8), suggesting that LAC and aPLs may have prognostic value for cerebrovascular events in COVID-19 patients [21].

### 1.3 von Willebrand Factor and Factor VIII in the blood

Other factors such as von Willebrand Factor (vWF) and Factor VIII (FVIII), were elevated in COVID-19 in a French study [6]. Escher et al. also reported a case with an increase in D-dimer, antiphospholipid-antibodies, vWF and FVIII [22]. Elevated vWF and FVIII in the blood suggest the presence of endothelial inflammation. Platelet adhesion mediated by vWF could lead to a hypercoagulation status [23].

Coagulation disorders also have important clinical implications. A meta-analysis suggested that PT and D-dimer levels were remarkably elevated in severe cases, but no significant changes were found in the platelet counts or activated partial thromboplastin time[24]. Although the traits of coagulation disorders in COVID-19 patients normally look like but are distinct from those of DIC, DIC occurs at a high frequency in severe cases with respiratory failure[9], which contributes to the organ failure caused by hemodynamic instability [14].

### 1.4 Possible role of plasma fibronectin

VWF and fibrinogen are important factors in blood clot formation. However, thrombus formation can still occur in the absence of these two factors [25, 26]. Plasma fibronectin (pFn) has been reported to support hemostasis and regulate thrombosis, which may also contribute to blood clot formation in COVID-19 [27]. pFn forms deposits and contributes to hemostasis even before platelet accumulation at the vessel injury site in a mouse model. When linked with fibrin, pFn can enhance platelet aggregation. The absence of fibrin will lead to an inhibition of the process. The study concluded that pFn is a supportive factor in hemostasis and becomes vital in coagulation deficiency cases. Therefore, it is reasonable and thought-provoking to consider pFn as a potential cause. There are also other possible important factors in the coagulation process in COVID-19, but more studies and research need to be done on this topic.

### 1.5 Age

COVID-19 is an age-related disease with a higher risk of organ dysfunction and mortality in older adults [28]. Generally, older people are more prone to thromboembolism because of enhanced coagulation activation and a ‘prethrombotic state’, which is caused by elevated coagulation activation peptide levels and a weaker fibrinolytic system[29]. It has even been reported that coagulation reference intervals are different in older people and age-related intervals may need to be further established [30]. A study showed that older COVID-19 patients are more susceptible to DVT [31, 32]. Aging-related factors contribute to thrombosis in COVID-19. More inflammatory cytokines are produced in older people as a result of inflammaging and an exacerbated inflammatory response could be generated [33]. Additionally, older patients are more likely to suffer from comorbid disorders, thus making cytokine storms easier to induce by SARS-CoV-2 [34]. Comorbidities have also been proven to be associated with the mortality rate. In general, congestive heart failure, dementia, chronic pulmonary disease, liver disease, renal disease, and metastatic solid tumors were conducive to higher mortality in COVID-19 patients [35]. Myocardial infarction and renal disease were linked with higher mortality rates among all age groups, but there were differences for different age groups [35]. Mild liver disease and any tumor were associated with higher odds of death for people younger than 50 [35]. For those between 50 and 69 years old, congestive heart failure, chronic pulmonary disease, moderate/severe liver disease, metastatic solid tumor, and AIDS/HIV all contributed to higher odds of death [35]. For people aged 70-90 years, congestive heart failure and dementia were to blame [35].

## 2. Thrombosis

Thrombosis in COVID-19 patients has been reported in several clinical studies (Table 2). A study was designed to identify the prevalence of venous thromboembolism (VTE) and found an occurrence of 25% (20/81) in severe COVID-19 patients [36]. A D-dimer cutoff value (>1.5 µg/mL) was established for VTE prediction, which had a sensitivity of 85.0% and a specificity of 88.5% [36]. Another study in 2 Dutch hospitals analyzed 184 ICU patients and showed that thrombosis occurred in multiple organs or systems and could cause complications such as PE, ischemic stroke, myocardial infarction, and systemic arterial embolism. The cumulative incidence of thrombotic complications was 31%, and VTE and arterial thrombotic events accounted for 27% and 3.7%, respectively. PE was the most frequent complication affecting 25 patients [37]. Patients with thrombotic complications are found to have a higher risk of all-cause death while no association was found between anticoagulation treatment and all-cause death [38].

Previous autopsy series have revealed more details about the development of thrombosis in COVID-19 patients. Pulmonary thrombosis was found in most cases undergoing autopsy because respiratory impairment and COVID-19 victims. It seems that small veins or capillaries are prone to thrombus formation due to the low velocity and narrow diameter of these small vessels. In pulmonary arteries at the hilum, gross findings showed no thromboemboli but did show a dark-colored hemorrhage with focal distinctions in the peripheral parenchyma. In some cases, small, firm thrombi occurred in areas of the peripheral parenchyma [39]. In microscopic findings, a diffuse alveolar damage was observed. Fibrinous thrombi were also detected in the small pulmonary arterioles of the peripheral parenchyma and a high risk of pulmonary microthrombosis in the small vessels, capillaries, and alveolar capillaries of the lungs was noticed in patients [40].

**Table 2:** Thrombosis in COVID-19 patients.

Thrombosis/organ involvement	Source	Country	Sample Size	Patients Type	Study Design	Findings
VTE	Cui et al [36]	China	81	ICU	Single-center, retrospective study	20 (25%) patients were diagnosed with VTE and 8 (10%) died. 1.5 µg/mL of D-dimer level was determined as a cutoff value in predicting VTE with a sensitivity of 85.0%, specificity of 88.5%, and NPV of 94.7%.
PE, DVT, ischemic stroke, MI, systemic arterial embolism	Klok et al [37, 38]	Nether lands	184	ICU	Multicenter, retrospective study	Coagulopathy was defined as prolongation of PT>3 s or APTT>5 s. Cumulative incidence was 31% in all patients. VTE accounted for 27% patients, and arterial thrombotic events for 3.7%. PE was the most common thrombotic complication. Patients with thrombotic complications had a higher risk of all-cause death.
Lung and heart	Fox et al [39]	US	4	Fatal	Autopsy series	Thrombosis and microangiopathy existed in the small vessels and capillaries of the lungs.
Lung and heart	Ueki et al [41]	Switzerland	1	Severe	Case report	STEMI and PE were complications in this COVID-19 patient.
Lung and kidney	Dolhnikoff et al [40]	Brazil	10	Fatal	Autopsy series	Fibrinous thrombi were found in small pulmonary arterioles of peripheral parenchyma in 8 cases, while glomeruli and superficial dermal vessels were mostly free of fibrinous thrombi.
Lung	Ackermann et al [52]	US	7	Fatal	Autopsy series	Alveolar capillary microthrombi in COVID-19 patients were 9 times more than in patients with influenza. Endothelial injury was found in the lungs.
Heart	Dominguez-Erquicia et al [42]	Spain	1	Emergency	Case report	STEMI was confirmed in a patient with no known cardiovascular risk factor.

Abbreviations: VTE, venous thromboembolism; NPV, negative predictive value; PE, pulmonary embolism; DVT, deep vein thrombosis; MI, myocardial infarction; STEMI, ST-elevation myocardial infarction. Note: VTE includes PE and DVT.

Other studies have shown that thrombosis also affects the heart. ST-elevation myocardial infarction (STEMI) and PE were reported in an older patient after SARS-CoV-2 infection [41]. A COVID-19 patient with no risk factors for cardiovascular disease was diagnosed with STEMI, and multivessel coronary thrombi were confirmed during coronary angiography [42]. Intriguingly, in three cases from New Orleans who underwent autopsies, the cardiac histopathologies did show myocyte necrosis but no thrombi formation were identified in the coronary arteries of any of the patients [39]. Pellegrini and his colleagues demonstrated that microthrombi were found in 64% of cases with cardiac injury. Microthrombi from patients with COVID-19 consist of fibrin II and complement complex C5b-9 [43]. Thrombotic complications in the heart are rarely recorded because of their life-threatening character. Differences in those reports may be explained by different stages or severity of the disease. Cardiomyocyte necrosis may also be caused by recurrent short periods of blockage of the bloodstream of the heart by migrating thrombi.

More convincing evidence came from a multicenter, prospective cohort study in France, which demonstrated that severe COVID-19 patients were at high risk of thrombosis. The study included 150 ICU patients from two centers in a French tertiary hospital. More thrombotic complications were observed in severe COVID-19 patients, and PE (n=25, 16.7%) had the highest frequency despite anticoagulation treatment. For those receiving continuous renal replacement therapy, 96.6% (28/29) of patients experienced circuit clotting, which may suggest an extra risk of hypercoagulation with some concomitant diseases [6].

## 3. Mechanism of the coagulation disorders and thrombosis

### 3.1 Endothelial cell injury in patients with SARS-CoV-2 infection

**Figure 1.**
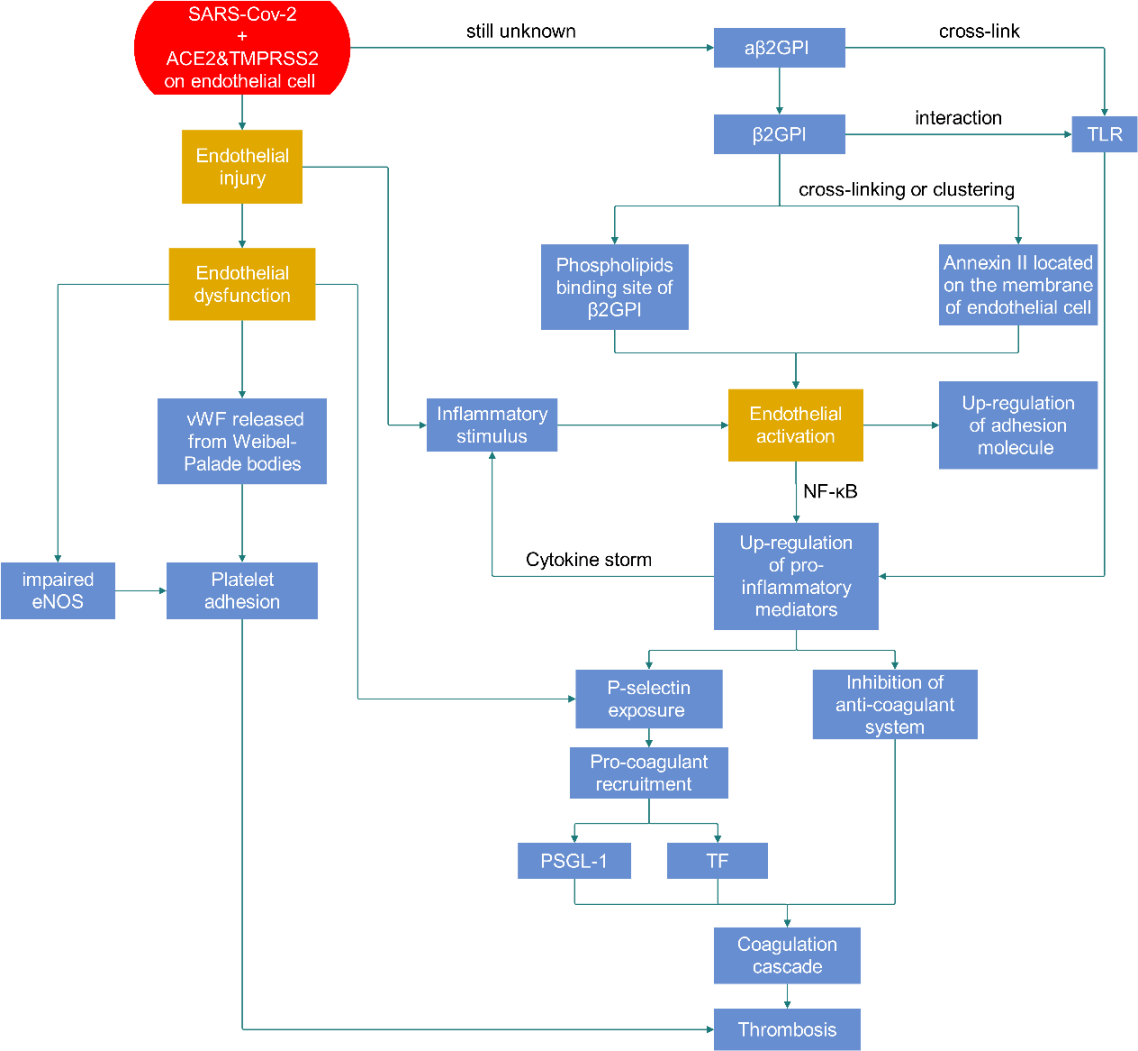
Thrombosis caused by endothelial dysfunction and activation.

Endothelial cell injury in COVID-19 patients may be a key step in causing coagulation disorders (Fig. 1). The endothelium is essential for normal coagulation function, and it is widely known that endothelial cell damage leads to vessel occlusion after triggering both intrinsic and extrinsic coagulation pathways. Angiotensin-converting enzyme 2 (ACE2) is recognized as the receptor of SARS-CoV-2 [2, 44]. ACE2 cleaves Ang II to Ang-(1-7) to maintain homeostasis in the renin-angiotensin-aldosterone system (RAAS). The entry of SARS-CoV-2 into infected cells is mainly mediated by ACE2 but is also facilitated by transmembrane protease serine type 2 (TMPRSS2) in S protein priming [45].

ACE2 is widely distributed in many organs, such as the lung, heart, kidney, liver, intestine and even the brain [46]. Recently, SARS-CoV-2 RNA or SARS-CoV-2-positive cells were detected in several organs in COVID-19 patients, including the nose, pharynx, lung, gut, heart, skeletal muscle, and bladder using a rhesus macaque model [47]. Clinically, organs expressing ACE2 suffer. Lung injury, acute cardiac injury and acute kidney injury are common in SARS-CoV-2 infected patients [48]. A lower prevalence of COVID-19 in the young may be explained partly by their lower ACE2 expression relative to that of adults [49].

Indirect evidence of endothelial injury comes from blood tests. vWF is stored in the Weibel-Palade bodies of endothelial cells and is released into the blood when the endothelium is damaged, so vWF is regarded as a marker (indirect evidence) of endothelial injury [50]. Elevated vWF activity (3 times to 4 times the upper limits of the normal range) in a COVID-19 patient suggests the presence of severe endothelial stimulation and damage [22].

Direct evidence was collected from pathological findings. Viral inclusion structures were demonstrated in endothelial cells from a transplanted kidney [51]. Lymphocytic endotheliitis has been demonstrated in the lung, heart, kidney, liver, and even the submucosal vessels of the small intestine [51]. Ackermann et al. examined lungs from patients who died of COVID-19 and compared them to those with acute respiratory distress syndrome (ARDS) caused by influenza A (H1N1). It was found that the endothelium was severely damaged by SARS-CoV-2 and that the membranes of endothelial cells were impaired [52]. The presence of endothelial cell injury, an indicator of the activation of the coagulation cascade, is also supported by viral cytopathic effects on the epithelium located in the alveolar and small airways, characterized by endothelial swelling as well as aggregated megakaryo-cytes in the pulmonary capillaries [53]. Therefore, viral inclusion structures and mRNA expression further supports that there is entry of SARS-CoV-2 into endothelial cells. Endotheliitis and swollen and damaged endothelial cells provide direct evidence for endothelial cell injury. All these findings indicate that endothelial cells are pathologically affected in COVID-19 patients. Both the invasion of SARS-CoV-2 via ACE2 and the inflammatory response contribute to endotheliitis in multiple organs. Apoptosis together with proptosis may also play a role in endothelial cell injury [51].

Although ACE2 is expressed on many organs/cells, we should note that some studies found that ACE2 is also expressed on the platelet surface and contributes to thrombosis by binding to SARS-CoV-2 spike protein[54]. However, this observation may need to be further confirmed since another study suggested that platelets are hyperactivated in the pathogenesis of COVID-19 [55]. The interaction between SARS-CoV-2 and platelets need to be demonstrated to illustrate the mechanism leading to hypercoagulative status [56].

### 3.2 Role of damaged endothelial cells in coagulation disorders and thrombosis

**Figure 2.**
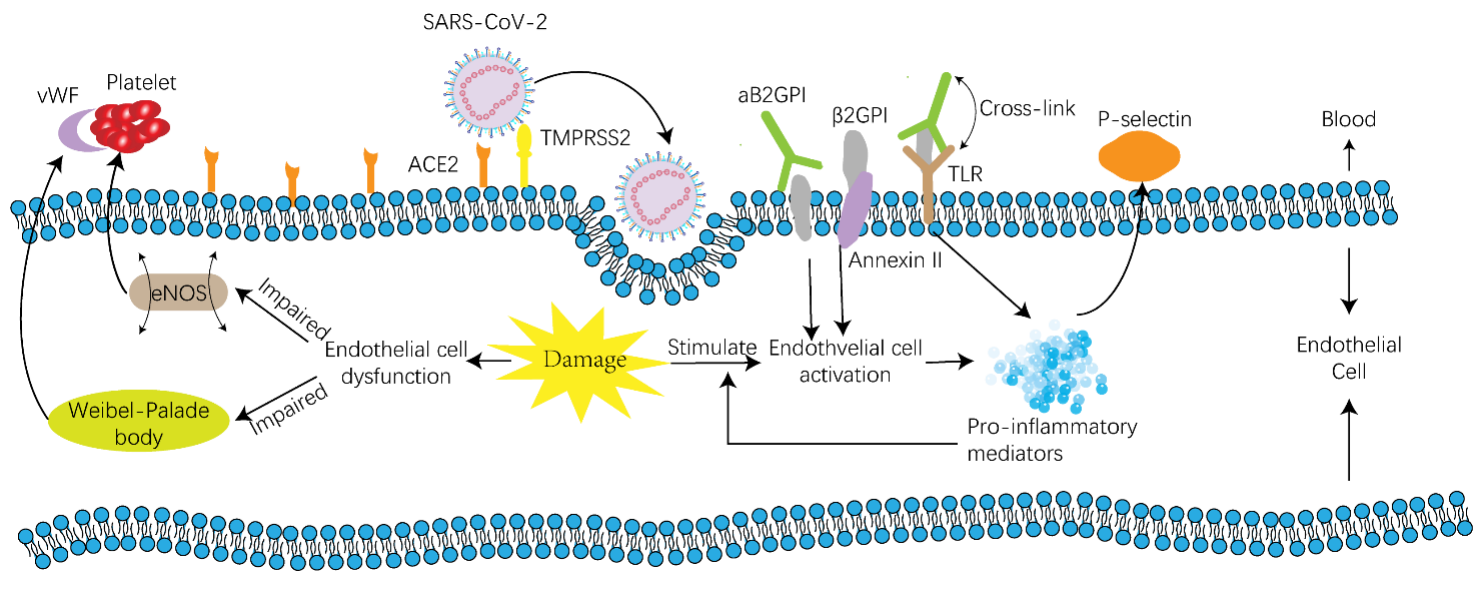
Mechanism of thrombosis on a cellular level. Endothelial dysfunction is the result of endothelial injury and will lead to clot formation because of platelet adhesion caused by vWF released from Weibel-Palade bodies and impaired eNOS. Endothelial cell activation can be induced by aβ2GPI, which belongs to aPLs. The upregulation of proinflammatory mediators and adhesion molecules on endothelial cells will occur after activation. Proinflammatory mediators can form a cytokine storm and contribute to a positive feedback loop in endothelial cell activation, leading to a hypercoagulation status and thrombosis. Adhesion molecules also attract platelets and play a role in thrombosis formation. Abbreviations: ACE2, angiotensin-converting enzyme 2; TMPRSS2, transmembrane protease serine type 2; eNOS, endothelial nitric oxide synthase; aβ2GPI, anti-β2-glycoprotein I antibodies, aPLs, antiphospholipid antibodies; TLR, Toll-like receptor; PSGL-1, P-selectin glycoprotein ligand-1; TF, tissue factor.

It is known that the endothelium can generate and secrete factors influencing the coagulation system such as heparin cofactor 2, factor V, factor VIII, protein S, protein C, thrombomodulin, tissue factor, vWF and plasminogen activator inhibitor [57, 58]. Endotheliopathy has been observed in SARS-CoV-2 patients [59]. COVID-19 has even been suspected to be an endothelial disease because of its complication profile, such as thrombosis, hypertension, renal failure, and diabetes [60]. In fact, both endothelial activation and dysfunction develop in COVID-19 patients and contribute to their coagulation disorders and thrombosis (Fig. 2).

#### 3.2.1 Endothelial cell activation

The concept of endothelial cell activation was first proposed in the 1980s and refers to the expression of activated antigens on the surface of endothelial cells being stimulated by inflammatory mediators [61]. Endothelial cell activation has a wider meaning, illustrating the response of endothelial cells to inflammatory stimuli, both in vivo and *in vitro* [62].

Endothelial cell activation can be triggered by inflammatory mediators in COVID-19 patients. Inflammatory mediators such as IL-6, TNFα and IL-8, are markedly elevated in some patients [63]. Such inflammatory responses are also found in severe acute respiratory syndrome (SARS) and Middle East respiratory syndrome (MERS), which can both lead to lung injury and even death [64].

In addition to the inflammatory response, endothelial cell activation can also be induced by aPLs, especially aβ2GPI, which targets β2GPI on endothelial cells. β2GPI may bind to endothelial cells through at least two mechanisms[65]. First, the fifth domain of β2GPI is presumed to be a phospholipid binding site because of its positive charge, thus enabling its insertion into the endothelial cell membrane bilayer [66]. A second mechanism involves the endothelial cell receptor Annexin II, which is surprisingly not a transmembrane protein but a receptor for tissue plasminogen activator (t-PA) [67]. Two procedures are induced after endothelial cell activation: upregulation of proinflammatory mediators and the expression of cell adhesion molecules. Upregulation of proinflammatory mediators is triggered through NF-κB translocation and Toll-like receptors. Molecular mimicry between β2GPI and pathogen structures contributes to the interaction between β2GPI and TLR, and TLR is triggered after cross-linking between aβ2GPI and TLR [68]. Inflammatory mediators generated in endothelial cell activation will enhance the activation process and form a positive feedback loop, and even a cytokine storm. A multicenter, retrospective study suggested that mortality may be attributed to a cytokine storm or to fulminant myocarditis [69].

Cell adhesion molecules are regulated via an Annexin II-mediated mechanism. β2GPI first binds to Annexin II with high affinity on inactivated endothelial cells [70]. Then, a signaling response is triggered after cross-linking or clustering, and endothelial cell activation occurs, which leads to the expression of endothelial cell adhesion molecules such as P-selectin [67]. Finally, procoagulant microparticles bearing tissue factor and P-selectin glycoprotein ligand-1 (PSGL-1) will aggregate and the coagulation cascade is triggered [71].

Moreover, inflammatory mediators such as IL-1 and TNF-α are known to regulate the functional phenotype of endothelial cells [72]. Leukocytes then migrate, which is followed by an increase in the vascular permeability, and then the formation of clots or a thrombus. Inflammatory mediators also enhance the expression of procoagulant tissue factor and at the same time inhibit the anticoagulant system [72], including tissue factor pathway inhibitor (TFPI), which is an inhibitor of the coagulation cascade that is generated by endothelial cells [57].

#### 3.2.2 Endothelial cell dysfunction

As the name suggests, endothelial cell dysfunction is a condition where endothelial cells fail to perform their normal function, including functioning as a barrier thus maintaining homeostasis, balancing clotting and initiating fibrinolysis [73]. Endothelial cell injury has been proven by both endothelial damage markers in the blood and direct pathological evidence in COVID-19 patients. Endothelial dysfunction-related thrombosis has been reported in patients with idiopathic VTE [73]. P-selectin and vWF are recognized as markers of endothelial damage and a long-term elevation of those markers is detected in DVT patients [74]. As a multimeric glycoprotein with multiple domains, vWF plays an important role in maintaining the balance between hemorrhage and clotting. We already know that platelet adhesion is mediated by vWF on damaged sites of the vascular system and that vWF also carries factor VIII in the circulation [75, 76]. Elevated vWF in the plasma of COVID-19 patients has been discussed and could contribute to the clotting process.

Endothelial dysfunction is also characterized by a modified endothelial function in the nitric oxide synthase (NOS) system, endothelial tension [57], and any other alteration of endothelial cells [77]. There are 3 kinds of genes regulating NO in the human body. They are neuronal NOS (nNOS), cytokine-inducible NOS (iNOS) and endothelial NOS (eNOS) [78]. eNOS generates endothelium-derived NO and is essential for endothelial function. It is known that endogenous NO has the ability to prevent platelets from adhering to the vascular endothelium [79]. Studies in humans and animals have proven the antithrombotic effects of eNOS, and NO is generated by endothelial cells and platelets[80]. Clinically, plasma nitrite levels are lower in patients with antiphospholipid syndrome, which is consistent with the impaired endothelial function [81].

To conclude, SARS- CoV-2 will cause both endothelial cell activation and dysfunction. Endothelial activation is caused by inflammatory mediators and will promote an inflammatory response as well as the production of pro-coagulants as positive feedback to form a cytokine storm. Endothelial dysfunction will lead to the release of vWF and the impairment of eNOS, and both contribute to platelet adhesion and result in thrombi.

Although endothelial cells play a significant role in coagulation disorders, there are other possible pathways leading to thrombosis. For example, Becker mentioned that neutrophil extracellular traps (NETs) play a role in clot formation by platelet-NET interactions [82].

## 4. Mechanism of coagulopathy in SARS and MERS

SARS was an epidemic caused by SARS-CoV-1 in 2003 and affected 26 countries. Coagulation disorders and thrombotic complications are also seen in patients with SARS [83]. Interestingly, ACE2 also acts as the receptor for SARS-CoV-1 [84]. SARS-CoV-1 infection leads to the overexpression of several genes, including the thromboxane synthase (TBXAS) gene modulating platelet aggregation and fibrin (specifically FGB and FGG) generation [85]. SARS-CoV-1 infection also upregulates genes in coagulation pathways such as factors II, III, X and SERPINs (D1 and A3). These genetic findings are consistent with the presence of thrombosis in small vessels at autopsy [86]. Endothelial dysfunction is caused by the overexpression of thromboxane [87].

MERS emerged in 2012 and is caused by MERS-CoV (HCoV-EMC/2012)[88]. In contrast to SARS-CoV-1 and SARS-CoV-2, entry of MERS-CoV is mediated by dipeptidyl peptidase 4 (DPP4, CD26), which is a type II transmembrane ectopeptidase [89]. For coagulation disorders, thrombocytopenia was reported in a retrospective study, where platelet levels were 164 ± 76.57×10^9^/L in MERS-CoV-positive patients versus 240 ± 79.87×10^9^/L in MERS-CoV-negative patients [90]. It was fatal in most patients once DIC developed [91]. The affected coagulation cascade with a MERS-CoV infection has been illustrated with a transgenic mouse expressing human DPP4 (hDPP4), and microthrombi and alveolar edema were found in the lungs while the airways were free of pathological changes [92].

## 5. Recognition and Management of Coagulopathy

Among the diverse parameters mentioned above, repeated tests of D-dimer, PT and platelet counts every 2 or 3 days are recommended for diagnosing coagulopathy in COVID-19 patients [10]. D-dimers, PT, and platelet count are also recommended as markers for evaluating coagulation status, among which D-dimer matters the most [93]. An algorithm was provided by Levi, which suggested that patients with a significantly elevated D-dimer (3-4 fold), a prolonged PT, a platelet count less than 100×10^9^/L or a fibrinogen level less than 2.0 g/L should be hospitalized and need close monitoring. These parameters are again arranged in descending order of importance [93]. The D-dimer level is of great importance since patients with high levels of D-dimer tend to require mechanical ventilation and are more prone to death. Thus, Bikdeli et al. suggested monitoring D-dimer levels, PT, platelet counts and fibrinogen levels [94]. However, there is some controversy regarding the safety in using those 4 markers for clinical decisions, and reliable clinical assessments instead are recommended [95]. LAC is associated with a prothrombotic state, especially in critically ill patients with a suspicion of stroke. It is suggested that LAC/Lps screening may be useful in selected high-risk patients.

Low molecular weight heparin (LMWH) should be considered in all hospitalized COVID-19 patients since elevated D-dimer levels will result in a high mortality rate [96]. In one study, 449 severe patients with COVID-19 were recruited and 99 were treated with LMWH for at least 7 days. It was found that LMWH benefits patients with high sepsis-induced coagulopathy (SIC) scores (≥4) or significantly increased D-dimer levels (>6 fold) [96]. Unfractionated heparin is also recommended [97, 98]. Appropriate treatment for coagulation disorders is required in COVID-19 patients [97]. A consensus from Chinese experts recommended both unfractionated heparin and LMWH without preferring one over the other[99]. Bikdeli et al. agreed with the ISTH interim guidelines and advocated for the use of LMWH in the treatment protocols[94]. In patients with cardiac events, Bikdeli proposed dual antiplatelet therapy (DAPT) and full dose anticoagulation for type I myocardial infarctions[94]. However, whether more intensive anticoagulation therapy or direct thrombin inhibitors should be used in severely ill patients with an existing risk of coagulopathy needs to be further investigated [100].

### Controversial points and future directions

Coagulation disorders are significant in COVID-19 patients with elevated D-dimer levels, a prolonged PT, and a decreased platelet count, which may cause thrombosis in severe cases. Endothelial cell activation and dysfunction may be the underlying mechanisms. Anti-coagulation treatment is recommended despite clinicians’ different preferences for LMWH or unfractionated heparin.

How aPLs are generated in COVID-19 patients is still unknown, and the prevalence of aPLs is controversial in COVID-19 patients. Connell et al. suggested that aPLs were common in infection, so the role of aPLs as the agent causing the thrombosis is problematic [101]. In contrast, Galeano-Valle et al. demonstrated that aPLs were not common in COVID-19 patients with thromboembolism. He found that only 2 patients (8.3%) out of a total of 24 were aPL positive [102]. Of note, the size of the patient population in Galeano-Valle’s study was limited, and there was a lack of serologic confirmation[102]. Differences in the frequency of aPLs may be influenced by the stage of the disease, race of the patients or extent of mutation of SARS-CoV-2. More evidence is needed to further our understanding of both the virus and our knowledge of coagulation disorders and their mechanism in COVID-19 patients.
